# Temperature during larval development and adult maintenance influences the survival of *Anopheles gambiae* s.s.

**DOI:** 10.1186/s13071-014-0489-3

**Published:** 2014-11-05

**Authors:** Céline Christiansen-Jucht, Paul E Parham, Adam Saddler, Jacob C Koella, María-Gloria Basáñez

**Affiliations:** Department of Infectious Disease Epidemiology, School of Public Health, Faculty of Medicine (St Mary’s campus), Imperial College London, Norfolk Place, London, W2 1PG UK; Division of Biology, Faculty of Life Sciences, Imperial College London, Silwood Park Campus, Ascot, SL5 2PZ Berkshire UK; Department of Public Health and Policy, Faculty of Health and Life Sciences, University of Liverpool, Liverpool, L69 3GL UK; Present address : Faculté des Sciences, Institut de Biologie, Université de Neuchâtel, Rue Emile-Argand 11, CH-2000 Neuchâtel, Switzerland; Grantham Institute for Climate Change, Department of Infectious Disease Epidemiology, School of Public Health, Faculty of Medicine, St. Mary’s campus, Imperial College London, London, W2 1PG UK

**Keywords:** *Anopheles gambiae sensu stricto*, Environmental temperature, Larval survival, Mosquito survival, Climate change

## Abstract

**Background:**

Malaria transmission depends on vector life-history parameters and population dynamics, and particularly on the survival of adult *Anopheles* mosquitoes. These dynamics are sensitive to climatic and environmental factors, and temperature is a particularly important driver. Data currently exist on the influence of constant and fluctuating adult environmental temperature on adult *Anopheles gambiae* s.s. survival and on the effect of larval environmental temperature on larval survival, but none on how larval temperature affects adult life-history parameters.

**Methods:**

Mosquito larvae and pupae were reared individually at different temperatures (23 ± 1°C, 27 ± 1°C, 31 ± 1°C, and 35 ± 1°C), 75 ± 5% relative humidity. Upon emergence into imagoes, individual adult females were either left at their larval temperature or placed at a different temperature within the range above. Survival was monitored every 24 hours and data were analysed using non-parametric and parametric methods. The Gompertz distribution fitted the survivorship data better than the gamma, Weibull, and exponential distributions overall and was adopted to describe mosquito mortality rates.

**Results:**

Increasing environmental temperature during the larval stages decreased larval survival (p < 0.001). Increases of 4°C (from 23°C to 27°C, 27°C to 31°C, and 31°C to 35°C), 8°C (27°C to 35°C) and 12°C (23°C to 35°C) statistically significantly increased larval mortality (p < 0.001). Higher environmental temperature during the adult stages significantly lowered adult survival overall (p < 0.001), with increases of 4°C and 8°C significantly influencing survival (p < 0.001). Increasing the larval environment temperature also significantly increased adult mortality overall (p < 0.001): a 4°C increase (23°C to 27°C) did not significantly affect adult survival (p > 0.05), but an 8°C increase did (p < 0.05). The effect of a 4°C increase in larval temperature from 27°C to 31°C depended on the adult environmental temperature. The data also suggest that differences between the temperatures of the larval and adult environments affects adult mosquito survival.

**Conclusions:**

Environmental temperature affects *Anopheles* survival directly during the juvenile and adult stages, and indirectly, since temperature during larval development significantly influences adult survival. These results will help to parameterise more reliable mathematical models investigating the potential impact of temperature and global warming on malaria transmission.

**Electronic supplementary material:**

The online version of this article (doi:10.1186/s13071-014-0489-3) contains supplementary material, which is available to authorized users.

## Background

Although historical data and theoretical models suggest that the distribution of malaria is much more sensitive to the scale-up of control measures than to climate change, it appears evident that climate change will affect the distribution and transmission of mosquito-borne diseases such as malaria [1] and thereby influence the extent to which the disease can be controlled. However, we currently have a limited understanding of how climatic factors affect the entomological parameters determining transmission. The most obvious question is how increasing temperatures associated with climate change will affect mosquito longevity and the duration of the parasite’s development within the mosquito, two of the most influential parameters underlying the transmission of mosquito-borne diseases.

However, temperature also shapes mosquito life-history traits that are associated with vector-competence and determines mosquito population density: a warmer environment leads to faster development and smaller adults. Mosquito size can influence epidemiologically relevant traits such as longevity, length of the gonotrophic cycle, immunocompetence, size of the bloodmeal (and probability of infection), biting rate, and intensity of infection. These traits in turn can affect mosquito survival [2] and parasite development [3]. The effect of temperature on mosquito life-history might also affect transmission by influencing fecundity, which is limited by size. Moreover, mosquito population density and fecundity feedback through larval density to influence the development of mosquitoes by density-dependent competition and mortality [4]. Although sketches of these interactions are known, their integration is lacking but essential to allow us to predict how temperature may influence malaria transmission. In this paper, we focus on the effect of temperature on mosquito survival.

Human malaria is transmitted via the bites of female *Anopheles* mosquitoes. Mosquitoes need to bite at least twice to acquire and transmit the infection, and the *Plasmodium* parasites undertake a complex sporogonic cycle within the vector, such that depending on environmental temperature, the duration of the extrinsic incubation period can be similar to the average life expectancy of the mosquito [5]. This makes malaria transmission particularly vulnerable to the daily survival probability of the vector, since it is necessary for mosquitoes to survive until completion of sporogony and beyond this in order to transmit salivary gland sporozoites to susceptible hosts.

*Anopheles* mosquitoes are sensitive to mean environmental temperature as well as its temporal fluctuations. Understanding how temperature influences vector ecology is therefore extremely important in predicting mosquito distribution as well as vector fitness and capacity to transmit malaria [6]. This understanding of the factors affecting vector populations will also improve projections of future malaria transmission, as environmental shifts due to climate change are likely to affect the global spread of malaria [7], and in particular, climatic factors that influence vector survival are likely to influence malaria transmission [8]. However, the magnitude of the vector population dynamics dependence on climatic factors remains uncertain [7-9].

Mosquito survival has been shown to depend on temperature, rainfall, and humidity [10], as well as other factors such as mosquito density [11,12], genetic diversity [13], and the ability to find a blood meal. Data have been reported on the influence of adult temperature on adult survival [14-18]. Fewer data exist on the influence of juvenile environmental temperature on juvenile survival [19-23], but none exist, to our knowledge, on the influence of environmental temperature during the juvenile stages on adult mortality, although temperature throughout the mosquito’s development may have repercussions on its survival [24].

This report presents the results of an experimental investigation into the influence of environmental temperatures during the *Anopheles* mosquito’s juvenile and adult stages on survival. It has been suggested that the maternal environment has an influence on the population dynamics of *Anopheles* mosquitoes through its impact on offspring development, survival, and susceptibility [25].

## Methods

### Larval maintenance and temperature regimes

*Anopheles gambiae sensu stricto* (*s.s.*) mosquitoes, originating from the Kisumu colony from Western Kenya, were maintained at Imperial College London’s Silwood Park campus. Two days after hatching, larvae were individually placed in 12-well plates with 3 mL of deionised water, at one of the following environmental (air) temperatures: 23 ± 1°C, 27 ± 1°C, 31 ± 1°C, and 35 ± 1°C. For every temperature, 640 larvae were reared at a food regime of TetraMin® baby fish food until development into imagoes. On day 2 after hatching, larvae were given 0.02 μg of baby fish food per 100 mL of de-ionised water; on days 3, 4, 5, and 6, they were given 0.06, 0.08, 0.16, and 0.32 μg per mL respectively; and on days 7 until pupation, 0.6 μg per mL.

Mosquitoes were reared in a 12:12 light/dark cycle, at 75% (±5%) relative humidity (RH). Larvae were checked every 24 hours to count the number of dead and live, and to construct life-tables.

As each larva was reared individually, each mosquito was considered an individual data point. Our data is therefore representative only of the mosquito colony used in this experiment, and confirmation of our results is ideally required in other mosquito species, experimental, and field settings.

### Adult maintenance and temperature regimes

**Figure 1:**
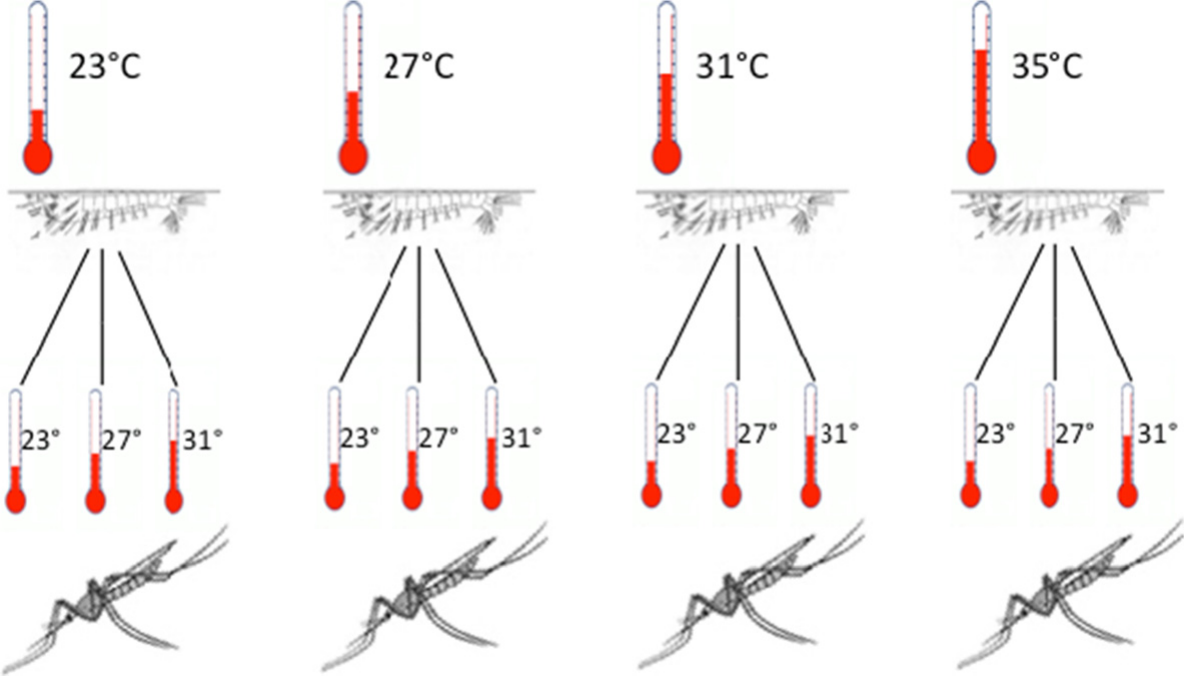
**Experimental design.** Larvae (640) reared at each temperature (23°C, 27°C, 31°C, 35°C) were allowed to develop into imagoes, and the adult females were kept at the same temperature at which they were reared as juveniles, or placed at one of the other two temperatures. None of the larvae reared at 35°C survived to adulthood, so no adults were maintained at that temperature.

Upon emergence into adults the mosquitoes were divided into three groups and each group was placed at either 23°C, 27°C, or 31°C (see Figure 1). This allowed the distinction between the effects of larval and adult environmental temperatures on adult survival. All larvae reared at 35°C died as immature stages and it was therefore decided not to maintain any adults at 35°C.

Adults were given four days to mate, before the females were placed in individual plastic cups and given a 10% sugar solution, while the males were discarded. Females were blood fed on CC-J’s arm on three occasions: 5, 12, and 19 days after emergence as adults. The time between blood meals was set as 7 days to allow all females to lay eggs (and the eggs to hatch). The sugar solution was removed 24 hours before each blood meal to ensure that females were eager to feed. Females that did not feed were discarded.

The bottom of each cup was filled with deionised water 24 hours after each blood meal to allow the females to lay eggs, and the mosquitoes were transferred to new, dry cups 48 hours after laying eggs. Adult survival was measured every 24 hours. All dead and live females were counted and the results recorded for the construction of life-tables. Censoring occurred 33 days after hatching, with all mosquitoes monitored until that day. On day 33, all mosquitoes still alive were frozen and their wing length measured.

In this report, only the survival data are presented. Data on larval developmental rates, adult female fecundity (number of eggs laid), fertility (number of eggs hatched), and mosquito size (measured by wing length) will be presented elsewhere.

### Statistical methods

#### Survival analysis

##### Non-parametric methods

Survival analyses were performed on each juvenile/adult temperature combination using Kaplan-Meier analysis [26], as this is a standard non-parametric method of representing survival data, and enables a useful comparison with data sets from similar experiments elsewhere to be made. The difference between results from different temperature regimens was compared using the log-rank and Mantel-Cox tests, both standard methods to test the null hypothesis that survival functions do not differ across groups. The log-rank test was used to compare the overall survival trend for the range of temperatures explored [27], and the Mantel-Cox test was used for two-sample comparisons of survivorship at one temperature against the survivorship at the baseline temperature (23°C) [27,28]. The results are given as a test statistic, which was compared with a Chi-squared distribution with one degree of freedom to yield a p-value. Mosquitoes killed on day 33 were classified as censored observations. The median survival time (with 95% confidence intervals) was calculated for each group to compare survival times, by determining the time beyond which 50% of the individuals in the population were expected to survive [27].

##### Parametric methods

In order to test the widely-applied assumption that adult *Anopheles* survival follows a model of constant mortality, the exponential, gamma, Gompertz, and Weibull survival functions [27] were fitted to larval and adult survival data at each temperature regimen by maximum likelihood estimation (MLE). The exponential model implies a constant mortality rate, whilst the remainder allow for age- (or time-) dependent mortality. In the log-likelihood function (log-*L*),

1 $$ \log L={\displaystyle \sum_t{N}_tS(t)+\left({N}_0-{N}_t\right)\left(1-S(t)\right)} $$

*N*_0_ is the number of mosquitoes (larvae or adults) alive at the beginning of the experiment, *N*_*t*_ is the number alive at the beginning of day *t*, and *S(t)* is the probability of surviving to day *t* according to the fitted survivorship function. Goodness-of-fit was compared using the Akaike Information Criterion (AIC), or by AICc (corrected Akaike Information Criterion) when the sample size was smaller than 80, to avoid over-fitting [29,30]. According to [29], a difference of ≤2 in AIC values indicates the two fits are not significantly different and only models with a difference of >4 in AIC values are statistically distinguishable. The Gompertz survival function,

2 $$ S(t)= \exp \left\{\frac{\uplambda}{\theta}\left[1- \exp \left(\theta t\right)\right]\right\} $$

was found to fit the survival data better than the exponential, gamma and Weibull survival functions in 10 out of 16 temperature scenarios, and was not significantly worse than the best fit in 2 further cases (Additional file 1: Table S1, Additional file 2: Table S2, Additional file 3: Table S3 and Additional file 4: Figure S1). This has implications for modelling *Anopheles* population dynamics and malaria transmission, as it suggests an age-dependent mortality model for the adult stages is more appropriate than assuming constant mortality. The best-fit Gompertz parameter values obtained by MLE at each environmental temperature were used in the corresponding hazard function

3 $$ h(t)=\uplambda \exp \left(\theta t\right) \exp \left\{\frac{\uplambda}{\theta}\left[1- \exp \left(\theta t\right)\right]\right\} $$

to describe larval and adult mortality at all temperatures regimes tested.

Uncertainty around the two Gompertz parameters at each larval and adult temperature was calculated using the profile likelihood method [31].

The non-parametric analyses were carried out using R, Version 2.10.1 (R Foundation for Statistical Computing, 2009), while Microsoft Excel (Microsoft Office 2008) was used for the parametric analyses and uncertainty calculations.

## Results

### The effect of larval environmental temperature on larval survival

**Figure 2:**
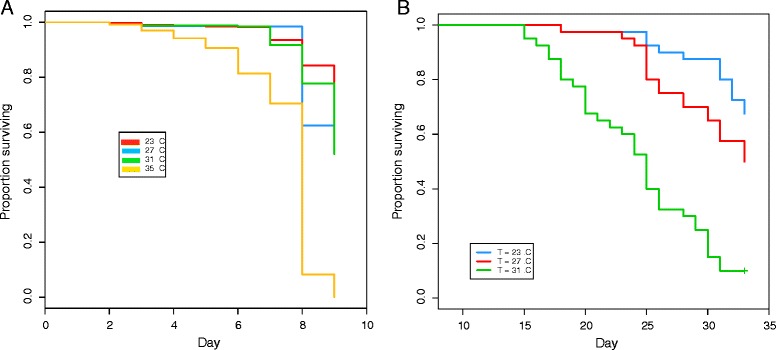
**Kaplan-Meier plots of** ***An. gambiae*** **larval (A) and adult (B) survival at different environmental temperatures.** The 23°C temperature (blue) was set as the baseline against which survival at other temperature was compared; 27°C (red); 31°C (green); 35°C (yellow).

Of the four larval groups, it was only possible to estimate the median survival time (8 days) for those reared at 35°C (Additional file 5: Table S4); for all lower temperatures, the survivorship curves did not cross the value of 0.5 (Figure 2A). According to the Kaplan-Meier plots, larval mortality increased notably with increasing environmental temperature (Figure 2A). The overall trend showed a statistically significant increase in mortality with increasing temperature (p < 0.001) (Additional file 6: Table S5). The decrease in larval survival was statistically significant for a 4°C increase in temperature (from 23°C to 27°C (p < 0.001)), as well as for an 8°C increase from 27°C to 35°C (p < 0.001), and a 12°C increase (from 23°C to 35°C, p < 0.001). The data resulting from an 8°C increase in environmental temperature from 23°C to 31°C did not allow us to perform a meaningful statistical test.

Decreases in larval survival were also statistically significant when the 4°C increases referred to temperatures other than the baseline; increases from 27°C to 31°C, and from 31°C to 35°C, both resulted in statistically significant increases in larval mortality with p < 0.001. All larvae reared at 35°C died before emergence into adults.

### The effect of adult environmental temperature on adult survival

Additional file 7: Table S6 and Additional file 8: Figure S2B indicate that, although the survivorship curve did not cross 0.5 for adult female mosquitoes maintained at 23°C, and it was therefore not possible to calculate the median survival time at this temperature, median survival decreased from 31 days (at 27°C) to 25 days (at 31°C). Overall, higher environmental temperatures were statistically and positively associated with an increase in adult mortality (p < 0.001) (Additional file 9: Table S7 and Figure 2B). The mortality experienced by adult mosquitoes was strongly and significantly more elevated with every increase in temperature relative to the baseline of 23°C, i.e. p-values were all highly significant (p < 0.001) for comparisons of 27°C vs. 23°C and 31°C vs. 27°C (each a 4°C increase), as well as for 31°C vs. 23°C (8°C increase, Additional file 9: Table S7).

### The effect of larval environmental temperature on adult survival

Table 1 summarises the median survival times for each group of adult temperatures and the temperatures at which these adults had been reared as larvae. In general, there is a trend for decreasing median survival times of adult females with increasing adult environmental temperature. Within each group, for similar larval and adult environmental temperatures, median survival times tend to be higher than when larvae and adults are maintained at more divergent temperatures; for instance, when both larvae and adults are exposed to 31°C, median survival time is 26 days, but only 22 days when the larvae had been reared at 23°C.

**Table 1:** **Median survival times of adult** ***An. gambiae*** **s.s. according to the temperature of the adult environment, and the temperature at which the larvae that developed into such adults had been reared**

**Adult temperature (°C)**	**Larval temperature (°C)**	**Total number of adults exposed at the start**	**Median adult survival (days) (95% C.I.)**
23 ± 1	23 ± 1	39	ND*
	27 ± 1	40	ND*
	31 ± 1	24	32.0 (30, ND)
27 ± 1	23 ± 1	40	33.0 (31, ND)
	27 ± 1	40	33.0 (31, ND)
	31 ± 1	40	28.5 (25, 30)
31 ± 1	23 ± 1	26	22.0 (19, 25)
	27 ± 1	40	25.0 (22, 28)
	31 ± 1	23	26.0 (25, 30)

*ND: Not determined. Median survival defines the time point at which the survivorship curve crosses 0.5, or at which 50% of the sample is expected to survive. In this case, the survival function did not cross 0.5, and the median survival cannot be calculated.

**Figure 3:**
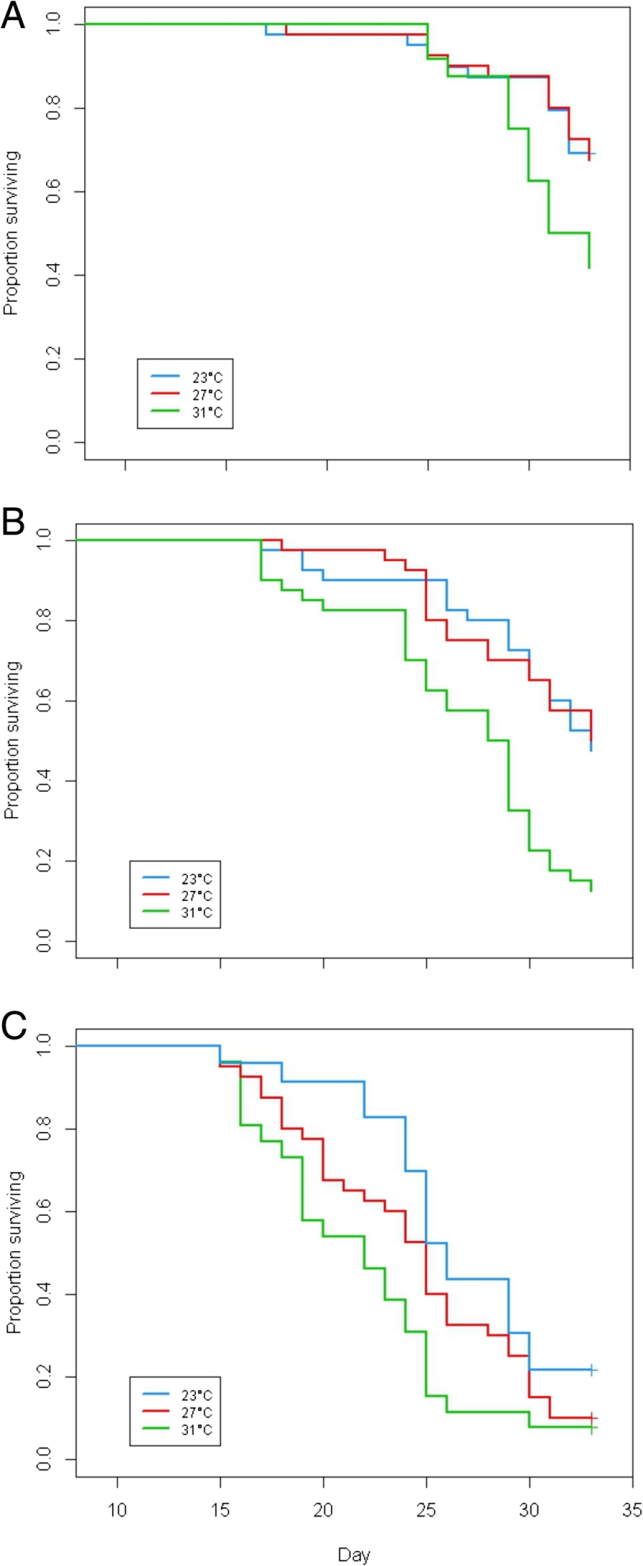
**Kaplan-Meier plots of** ***An. gambiae*** **adult survival at different environmental temperatures having been reared as larvae at different temperatures. (3A)**. Adult survival curves at adult environmental temperature of 23°C. Larval temperature 23°C (blue) was set as the baseline against which survival at other larval temperatures was compared; 27°C (red); 31°C (green). **(3B)**. Adult survival curves at adult environmental temperature 27°C. Larval temperature 23°C (blue) was set as the baseline against which survival at other larval temperatures was compared; 27°C (red); 31°C (green). **(3C)**. Adult survival curves at adult environmental temperature 31°C. Larval temperature 23°C (blue) was set as the baseline against which survival at other larval temperatures was compared; 27°C (red); 31°C (green).

Environmental temperature during the larval stages was found to have a marked effect on the survival of adult mosquitoes. When the mosquitoes reared at different temperatures were placed at 23°C as adults, those who had been reared at 27°C as larvae did not experience a significantly higher mortality than those reared at a larval temperature of 23°C (p = 0.92). However, those mosquitoes who were reared at 31°C had a higher mortality than larvae reared at 27°C (p < 0.05), and those mosquitoes that had been exposed to an 8°C decrease (from 31°C to 23°C) suffered a statistically significant increase in mortality compared to 23°C (p < 0.05) (Figure 3A and Table 2).

**Table 2:** **Two-group comparisons and overall trend of the effect of increasing larval environmental temperature on the survival of adult** ***An. gambiae*** **s.s. mosquitoes, at different adult environmental temperatures**

		**Larval temperature (°C)**			**Overall effect of larval temperature on adult survival**	
**Adult temperature (°C)**	**Test statistic**	**27 ± 1 (with respect to 23°C)**	**31 ± 1 (with respect to 23°C)**	**31 ± 1 (with respect to 27°C)**	**Test statistic**	
**23 ± 1**	Mantel-Cox test	0.01	4.88	4.63	Log-rank test	6.51
	p-value	0.920	0.027	0.031	p-value	0.039
**27 ± 1**	Mantel-Cox test	0.01	16.29	19.43	Log-rank test	23.51
	p-value	0.927	<0.001	<0.001	p-value	<0.001
**31 ± 1**	Mantel-Cox test	2.74	7.41	1.78	Log-rank test	7.61
	p-value	0.098	0.006	0.182	p-value	0.022
**All adult temperatures**					Log-rank test	108.30
					p-value	<0.001

When larvae reared at different temperatures (23, 27, 31°C) were moved to 27°C as adults, those who had also been reared at 27°C did not experience a significant decrease in adult survival compared with those reared at 23°C (p = 0.927), while those exposed to a 4°C decrease between the larval and the adult stages (from 31°C as juveniles to 27°C as adults) experienced a statistically significant reduction in adult survival (p < 0.001) (Figure 3B and Table 2).

Finally, when adults were kept at 31°C, mosquitoes experiencing a 4°C increase in temperature (from 27°C to 31°C) were not observed to have a significantly affected survival (p = 0.182), but an 8°C increase (from 23°C to 31°C) significantly increased adult mortality (p < 0.01). The overall influence of larval temperature on adult survival was significant when adults were maintained at 23°C (p < 0.05), 27°C (p < 0.001), and 31°C (p < 0.05) (Figure 3C), while the overall effect of larval environmental temperature on adult survival, at all adult temperatures, was highly significant (p < 0.001) (Table 2).

### Parametric curve fitting to survival and mortality data

Additional file 8: Figure S2 and Additional file 10: Figure S3 show the best-fit Gompertz survival curves for each combination of larval and adult temperatures. The values for the two parameters of the Gompertz survival function, λ and θ, and their 95% confidence intervals (CI) at each temperature are shown in Additional file 11: Figure S4. These values were used to develop Gompertz hazard functions, which were plotted against the mortality data for each temperature regimen (Additional file 12: Figure S5 and Additional file 13: Figure S6). Additional file 13: Figures S6a and S6b show that both parameters of the Gompertz survival functions vary widely as a function of larval temperature, whereas θ only changes significantly with respect to adult environmental temperature. Additional file 13: Figure S6c shows that for the Gompertz curves describing adult survival at adult temperatures of 23°C and 27°C, both λ and θ remain relatively invariant with respect to the temperature at which the larvae were reared, but change with larval environment temperature when adults were maintained at 31°C.

## Discussion

This study demonstrates that environmental temperature affects the survival of *Anopheles gambiae* s.s., both during their immature stage development and during their lifetime as adults. Results of the larval survival experiments indicated a statistically significant decrease in larval survival with every 4°C increase in environmental temperature, in agreement with previous studies [19,20,32]. Similarly, there was a statistically significant decrease in adult survival with each 4°C increase in environmental temperature, as has also been reported elsewhere [23,33,34].

However, this is the first study to investigate the effect of larval temperature on adult *Anopheles gambiae* survival. Our results indicate that a small difference (4°C) between the larval and adult temperatures may have a significant impact on adult survival, and this may depend on the temperature at which this difference occurs. This suggests that the temperature of the larval environment may have a much more important impact on the adult stages than was previously thought. Due to the complexities of the experimental setup and logistical constraints as to the number of mosquitoes that could be reared and observed, only four temperature treatments and only 4°C increases were investigated here. It is, therefore, difficult to extrapolate our conclusions to more nuanced increases in temperature.

In general, the Gompertz survivorship function fitted the survival data reasonably well, confirming the results of Clements and Paterson [35] and indicating the operation of age-dependent mortality (senescence) in both immature and adult stages, at least under laboratory conditions. Dawes *et al*. also reported age-dependent mortality in laboratory adult populations of *An. stephensi* [2]. Mosquito senescence has been documented in *Aedes aegypti*, both under laboratory and semi-field conditions [36,37]. As pointed out by other authors [35,36,38-40], vector-borne disease models tend to dismiss evidence supporting age-dependent vector mortality [41] for the sake of tractability, and because of contradictory evidence between laboratory and field studies [2,5,42], often assuming a constant hazard (and hence an exponential distribution of survival times, shown to give a poor fit to our data) [43]. In addition, our data suggest that age-dependent mortality in the juvenile and adult stages of *Anopheles gambiae* s.s. mosquitoes may depend on environmental temperature.

The results presented here give a detailed picture of larval survival at a range of temperatures. Previous studies have examined the effect of temperature on larval mortality rates (19), the percentage of larvae surviving to adulthood [20,23], the combined effect of larval density and temperature on survival rates [44], the combined effect of inter-species competition and temperature on the proportion of larvae developing to adults [23], and the effect of altitudinal changes in temperature on the proportion of larvae developing to adults [14]. Our study is, to our knowledge, the first to follow *Anopheles gambiae* s.s. larvae during their entire lifecycle.

Previous research into adult mortality has examined the probability of daily survival within a range of temperatures from 5°C to 40°C and with a humidity range from 40% to 100% [17,18], the different mortalities of emerging males and females [33], the time to 50% survival at different temperatures [45,46], the proportion surviving after exposure to high temperatures [16], and survival at different combinations of temperature and relative humidity (RH) [29]. However, our study differs from these by allowing mosquitoes to blood-feed and oviposit, mimicking more closely their true fate as adults.

We informally compare our results on adult mortality at a larval temperature of 27°C and adult temperatures of 23°C, 27°C, and 31°C, with 75 ± 5% RH, with those reported by Bayoh and Lindsay [Unpublished pers. comm.] at 20°C, 25°C, and 30°C, with 80% RH, and find (by visual inspection) similar survival curves (Additional file 14: Figure S7). The increased mortality in our study is likely due to the difference in experimental protocol. Host-seeking, blood feeding and oviposition carry a fitness and survival cost, using metabolic vector resources (allocated to reproduction), incurring a risk of drowning while laying, and placing a stress associated with travelling and displacement [47]. Inter-study differences in malaria vector survival as measured in captivity can be, partly, associated with allowing or not further blood-meals and egg-laying events (Heather Ferguson, pers. comm.).

It appears that the degree of influence of *An. gambiae* larval temperature on adult survival is dependent on adult temperature itself. Further experimentation is needed to determine whether a threshold exists above which increasing larval temperature significantly reduces adult survival, or whether an increase in larval temperature of a certain magnitude will only affect adult survival within certain environmental temperature margins. However, these hypotheses do not take into account temperature fluctuations between day and night, or diurnal fluctuations more generally.

The final draft of the Fifth Assessment Report by the Intergovernmental Panel on Climate Change [48] for the near-term climate (2016–2035) suggests that the global mean surface air temperature will increase by approximately 0.3°C–0.7°C, and that there will be an increase in the duration, intensity, and spatial reach of heat-waves. In light of the results presented here, which indicate that small changes in temperature are less likely to affect survival than larger fluctuations, the predicted short-term changes in temperature may not strongly influence *An. gambiae s.s.* distribution in areas where this vector is already established and present. Air temperatures currently vary broadly across Africa, with night-time air temperatures ranging from 6°C to 29.5°C, and daytime air temperatures from 17°C to 41.3°C [49]. This implies that the sensitivity of *An. gambiae* s.s. to changes in environmental temperature will be extremely region-specific.

Our experimental design did not take into account temperature fluctuations or differences in humidity that would affect mosquito development and survival in the field. Further investigation is needed to examine the effects of other climatic and environmental factors on *An. gambiae* survival and development rates. More research is also needed into the influence of local air temperature fluctuations and how these affect the temperature of the water in mosquito breeding sites. In addition, *Anopheles gambiae* s.s. is only one of seven dominant vector species of human malaria on the African continent [50], and data regarding the sensitivity of these other species to temperature and other climate- and population-related factors are equally sparse, if not more so. Climate change is likely to influence the survival [51] and life-history parameters of different species of malaria vectors in different manners [52]. More extensive, species-specific data on the dependency of mosquito life-history parameters and population dynamics on climatic conditions, when coupled with geographically-detailed climate predictions, will enable more robust and reliable predictions of vector population dynamics and disease transmission.

## Conclusions

Climate change is expected to lead to global and regional changes in environmental temperature and other climatic variables [48], which are likely to have an impact on vector distribution in sub-Saharan Africa and other malaria-prone regions [49,51]. It is thought that global warming may make currently inhospitable regions amenable to vector expansion along altitudinal gradients [1]. In order to generate useful predictions of malaria transmission and the impact of intervention programmes, the full impact of environmental conditions on the life-history parameters and population dynamics of disease vectors needs to be taken into account when forecasting transmission.

Our data show that the environmental temperatures to which *Anopheles gambiae s.s.* mosquitoes are exposed during both the juvenile and adult stages significantly affect the survival of this malaria vector both directly and indirectly, as temperatures during larval development influence adult survival. The direct effect of environmental temperature on larval and adult survival is highly significant for the range explored (23°C to 35°C), as it is for almost all temperature increases investigated.

We document here for the first time that the temperature to which *Anopheles gambiae* s.s. larvae are exposed during their development also influences the mortality of the adult females. This may have important implications for *Anopheles* population dynamics and ecology, and the diseases these mosquitoes transmit. Our results also show that the Gompertz distribution fits data on adult *Anopheles gambiae* survival in the laboratory significantly better than other parametric functions, including the exponential, implying that *Anopheles gambiae* mortality in the laboratory is age-dependent. This needs further confirmation from mortality data in the field, as evidence of age-dependent mortality has important implications for modelling vector population dynamics and the spread of malaria, requiring re-assessment of the common assumption in vector and transmission models that adult mosquito mortality does not depend on age.

## Additional files

Additional file 1: Table S1. Akaike Information Criterion (AIC) values for the exponential, gamma, Gompertz, and Weibull fits to larval survival data (* indicates the best fit). Additional file 2: Table S2. Akaike Information Criterion (AIC) values for the exponential, gamma, Gompertz, and Weibull fits to adult survival data (* indicates the best fit). Additional file 3: Table S3. AICc values for the exponential, gamma, Gompertz, and Weibull fits to adult survival data, subdivided by larval temperature (* indicates the best fit, ^‡^ indicates where the Gompertz fit is not significantly worse than the best fit). Additional file 4: Figure S1. Parametric fitting. An example of the fitting of four parametric survival functions (exponential (yellow), Gompertz (green), gamma (red), and Weibull (blue)) to larval survival data at environmental temperature 35°C. Additional file 5: Table S4. Median survival times of *An. gambiae* s.s. larvae at different environmental temperatures. *ND: Not determined. Median survival defines the time point at which the survivorship curve crosses 0.5, or at which 50% of the sample is expected to survive. In this case, the survival function did not cross 0.5, and the median survival cannot be calculated. Additional file 6: Table S5. Two-group comparisons and overall trend of the effect of larval environmental temperature on *An. gambiae* s.s. larval survival. *The comparison between 31°C and 23°C generated partly indistinguishable data, which did not allow us to perform a meaningful statistical test. Additional file 7: Table S6. Median survival times of *An. gambiae* s.s. adults at different environmental temperatures. *ND: Not determined. Median survival defines the time point at which the survivorship curve crosses 0.5, or at which 50% of the sample is expected to survive. In this case, the survival function did not cross 0.5, and the median survival cannot be calculated. Additional file 8: Figure S2. Gompertz fits to larval survival data. The Gompertz survival functions (red) are shown alongside the larval survival data at all environmental temperatures (23°C, 27°C, 31°C, and 35°C) to which they were fitted. Additional file 9: Table S7. Two-group comparisons and overall trend of the effect of adult environmental temperature on *An. gambiae* s.s. adult survival. Additional file 10: Figure S3. Gompertz fits to adult survival data. **(A)**. The Gompertz survival functions (red) are shown alongside the adult survival data at all adult temperatures (23°C, 27°C, 31°C) to which they were fitted. **(B)**. The Gompertz survival functions (red) are shown alongside the adult survival data at all combinations of larval and adult temperatures to which they were fitted. Additional file 11: Figure S4. Values of the Gompertz survival function parameters, λ and *θ*. **(A)**. Parameters for the Gompertz survival function fitted to the larval survival data at each larval temperature are shown with their 95% confidence intervals (CI). **(B)**. Parameters for the Gompertz survival function fitted to the adult survival data at each adult temperature are shown with their 95% CI. (C). Parameters for the Gompertz survival function fitted to the adult survival data at each combination of larval and adult temperatures are shown with their 95% CI. Additional file 12: Figure S5. Best-fit Gompertz survival function plotted against larval survival data. The Gompertz functions (blue) are shown alongside the larval survival data at all environmental temperatures (23°C, 27°C, 31°C, and 35°C). Additional file 13: Figure S6. Gompertz survival function plotted alongside adult mortality data. **(A)**. The Gompertz functions (blue) are shown alongside the adult mortality data at all adult temperatures (23°C, 27°C, 31°C). **(B)**. The Gompertz functions (blue) are shown alongside the adult mortality data at all combinations of larval and adult temperatures. Additional file 14: Figure S7. Comparison of survival curves with those generated by Bayoh and Lindsay (30). **(A)**. Survival curves by Bayoh and Lindsay, larval temperature 26°C, 80% RH. Adult survival at environmental temperature 20°C (yellow), 25°C (orange), and 30°C (red). **(B)**. Survival curves with data from this study, larval temperature 27°C, 75% RH. Adult survival at environmental temperature 23°C (yellow), 27°C (orange), 31°C (red).

## Abbreviations

AIC: Akaike Information Criterion
AICc: Corrected Akaike Information Criterion
CI: Confidence Intervals
MLE: Maximum Likelihood Estimation
RH: Relative humidity
s.s.: Sensu stricto

## References

[1] Siraj AS, Santos-Vega M, Bouma MJ, Yadeta D, Carrascal DR, Pascual M (2014). Altitudinal changes in malaria incidence in highlands of Ethiopia and Colombia. Science.

[2] Dawes EJ, Churcher TS, Zhuang S, Sinden RE, Basáñez M-G (2009). Anopheles mortality is both age- and Plasmodium-density dependent: implications for malaria transmission. Malar J.

[3] Churcher T, Bousema T, Walker M, Drakeley C, Schneider P, Ouédraogo A, Basáñez M-G (2013). Predicting mosquito infection from Plasmodium falciparum gametocyte density and estimating the reservoir of infection. eLife.

[4] White MT, Griffin JT, Churcher TS, Ferguson NM, Basáñez M-G, Ghani AC (2011). Modelling the impact of vector control interventions on Anopheles gambiae population dynamics. Parasit Vectors.

[5] Bellan SE (2010). The importance of age dependent mortality and the extrinsic incubation period in models of mosquito-borne disease transmission and control. PLoS One.

[6] Semenza JC, Menne B (2009). Climate change and infectious diseases in Europe. Lancet Infect Dis.

[7] Sutherst RW (2004). Global change and human vulnerability to vector-borne diseases. Clin Microbiol Rev.

[8] Craig M, Snow R, le Sueur D (1999). A climate-based distribution model of malaria transmission in sub-Saharan Africa. Parasitol Today.

[9] Lafferty K (2009). The ecology of climate change and infectious diseases. Ecology.

[10] Warrell D, Gilles H (2002). Essential Malariology.

[11] Gilles JRL, Lees RS, Soliban SM, Benedict MQ (2011). Density-dependent effects in experimental larval populations of Anopheles arabiensis (Diptera: Culicidae) can be negative, neutral, or overcompensatory depending on density and diet levels. J Med Entomol.

[12] Muriu SM, Coulson T, Mbogo CM, Godfray HCJ (2013). Larval density dependence in Anopheles gambiae s.s., the major African vector of malaria. J Anim Ecol.

[13] Tchuinkam T, Simard F, Lélé-Defo E, Téné-Fossog B, Tateng-Ngouateu A, Antonio-Nkondjio C, Mpoame M, Toto J-C, Njiné T, Fontenille D, Awono-Ambéné H-P (2010). Bionomics of Anopheline species and malaria transmission dynamics along an altitudinal transect in Western Cameroon. BMC Infect Dis.

[14] Afrane YA, Zhou G, Lawson BW, Githeko AK, Yan G (2007). Life-table analysis of Anopheles Arabiensis in Western Kenya Highlands: effects of land covers on larval and adult survivorship. Am J Trop Med Hyg.

[15] Afrane YA, Zhou G, Lawson BW, Githeko AK, Yan G (2006). Effects of microclimatic changes caused by deforestation on the survivorship and reproductive fitness of Anopheles gambiae in western Kenya highlands. Am J Trop Med Hyg.

[16] Kirby MJ, Lindsay SW (2004). Responses of adult mosquitoes of two sibling species, Anopheles arabiensis and A. gambiae s.s. (Diptera: Culicidae), to high temperatures. Bull Entomol Res.

[17] Midega JT, Mbogo CM, Mwnambi H, Wilson MD, Ojwang G, Mwangangi JM, Nzovu JG, Githure JI, Yan G, Beier JC (2007). Estimating dispersal and survival of Anopheles gambiae and Anopheles funestus along the Kenyan coast by using mark-release-recapture methods. J Med Entomol.

[18] Olayemi IK, Ande AT (2008). Survivorship of Anopheles gambiae in relation to malaria transmission in Ilorin, Nigeria. Online J Health Allied Sci.

[19] Bayoh MN, Lindsay SW (2004). Temperature‐related duration of aquatic stages of the Afrotropical malaria vector mosquito Anopheles gambiae in the laboratory. Med Vet Entomol.

[20] Bayoh MN, Lindsay SW (2003). Effect of temperature on the development of the aquatic stages of Anopheles Gambiae Sensu Stricto (Diptera: Culicidae). Bull Entomol Res.

[21] Huang J, Walker ED, Vulule J, Miller JR (2006). Daily temperature profiles in and around Western Kenyan larval habitats of Anopheles gambiae as related to egg mortality. Malar J.

[22] Impoinvil DE, Cardenas GA, Gihture JI, Mbogo CM, Beier JC (2007). Constant temperature and time period effects on Anopheles Gambiae egg hatching. J Am Mosq Control Assoc.

[23] Kirby MJ, Lindsay SW (2009). Effect of temperature and inter-specific competition on the development and survival of Anopheles gambiae sensu stricto and An. arabiensis larvae. Acta Trop.

[24] Beck-Johnson L, Nelson W, Paaijmans K, Read A, Thomas M, Bjørnstad O (2013). The effect of temperature on anopheles mosquito population dynamics and the potential for malaria transmission. PLoS One.

[25] Vogels CBF, Bukhari T, Koenraadt CJM (2014). Fitness consequences of larval exposure to Beauveria bassiana on adults of the malaria vector Anopheles stephensi. J Invertebr Pathol.

[26] Kaplan E, Meier P (1958). Nonparametric estimation from incomplete observations. J Am Stat Assoc.

[27] Collett D (2003). Modelling Survival Data in Medical Research.

[28] Mantel N, Haenszel W (1959). Statistical aspects of the analysis of data from retrospective studies of disease. J Natl Cancer Inst.

[29] Bolker B (2008). Chapter 6: Likelihood and all that. Ecological Models and Data in R.

[30] Burnham K, Anderson D (2002). Model Selection and Multimodel Inference: A Practical Information-Theoretic Approach.

[31] McCallum H (2000). Population Parameters: Estimation for Ecological Models.

[32] Depinay J-MO, Mbogo CM, Killeen G, Knols B, Beier J, Carlson J, Dushoff J, Billingsley P, Mwambi H, Githure J, Touré AM, McKenzie FE (2004). A simulation model of African Anopheles ecology and population dynamics for the analysis of malaria transmission. Malar J.

[33] Armstrong JA, Bransby-Williams WR (1961). The maintenance of a colony of Anopheles gambiae, with observations on the effects of changes in temperature. Bull World Health Organ.

[34] Lunde T, Balkew M, Korecha D, Gebre-Michael T, Massebo F, Sorteberg A, Lindtjørn B (2013). A dynamic model of some malaria-transmitting anopheline mosquitoes of the Afrotropical region. II. validation of species distribution and seasonal variations. Malar J.

[35] Clements A, Paterson G (1981). The analysis of mortality and survival rates in wild populations of mosquitoes. J Appl Ecol.

[36] Styer L, Minnick S, Sun A, Scott T (2007). Mortality and reproductive dynamics of Aedes aegypti (Diptera: Culicidae) fed human blood. Vector-Borne Zoonotic Dis.

[37] Harrington L, Françoisevermeylen, Jones J, Kitthawee S, Sithiprasasna R, Edman J, Scott TW (2008). Age-dependent survival of the dengue vector Aedes aegypti (Diptera: Culicidae) demonstrated by simultaneous release-recapture of different age cohorts. J Med Entomol.

[38] Hancock P, Thomas M, Godfray HC (2009). An age-structured model to evaluate the potential of novel malaria-control interventions: a case study of fungal biopesticide sprays. Proc R Soc B Biol Sci.

[39] Brady OJ, Johansson MA, Guerra CA, Bhatt S, Golding N, Pigott DM, Delatte H, Grech MG, Leisnham PT, Maciel-de-Freitas R, Styer LM, Smith DL, Scott TW, Gething PW, Hay SI (2013). Modelling adult Aedes aegypti and Aedes albopictus survival at different temperatures in laboratory and field settings. Parasit Vectors.

[40] Parham P, Pople D, Christiansen-Jucht C, Lindsay S, Hinsley W, Michael E (2012). Modeling the role of environmental variables on the population dynamics of the malaria vector Anopheles gambiae sensu stricto. Malar J.

[41] Yang HM, Macoris MLG, Galvani KC, Andrighetti MTM, Wanderley DMV (2009). Assessing the effects of temperature on the population of Aedes aegypti, the vector of dengue. Epidemiol Infect.

[42] Ferguson H, Read A (2002). Why is the effect of malaria parasites on mosquito survival still unresolved?. Trends Parasitol.

[43] Wilson DL (1994). The analysis of survival (mortality) data: fitting Gompertz, Weibull, and logistic functions. Mech Ageing Dev.

[44] Lyimo EO, Takken W, Koella J (1992). Effect of rearing temperature and larval density on larval survival, age at pupation and adult size of Anopheles gambiae. Entomol Exp Appl.

[45] Lardeux F, Tejerina R, Quispe V, Chavez T (2008). A physiological time analysis of the duration of the gonotrophic cycle of Anopheles pseudopunctipennis and its implications for malaria transmission in Bolivia. Malar J.

[46] Boyd M (1949). Epidemiology of malaria: factors related to the definitive host; section IV: intermediate host. Malariol.

[47] Lyimo I, Ferguson H (2009). Ecological and evolutionary determinants of host species choice in mosquito vectors. Trends Parasitol.

[48] IPCC (2013). Working Group 1 Contribution to the IPCC Fifth Assessment Report. Climate change 2013: the Physical Science Basis.

[49] Garske T, Ferguson N, Ghani A (2013). Estimating air temperature and its influence on malaria transmission across Africa. PLoS One.

[50] Sinka M, Bangs M, Manguin S, Rubio-Palis Y, Chareonviriyaphap T, Coetzee M, Mbogo CM, Hemingway J, Patil AP, Temperley WH, Gething PW, Kabaria CW, Burkot TR, Harbach RE, Hay SI (2012). A global map of dominant malaria vectors. Parasit Vectors.

[51] Lyons C, Coetzee M, Chown S (2013). Stable and fluctuating temperature effects on the development rate and survival of two malaria vectors, Anopheles arabiensis and Anopheles funestus. Parasit Vectors.

[52] Sinka M, Bangs M, Manguin S, Coetzee M, Mbogo C, Hemingway J, Patil AP, Temperley WH, Gething PW, Kabaria CW, Okara RM, van Boeckel T, Godfray HCJ, Harbach RE, Hay SI (2010). The dominant Anopheles vectors of human malaria in Africa, Europe and the Middle East: occurrence data, distribution maps and bionomic précis. Parasit Vectors.

