# Supplementary material for: Temperature during larval development and adult maintenance influences the survival of Anopheles gambiae s.s
Source: Parasit Vectors. 2014 Nov 5;7:489. doi: 10.1186/s13071-014-0489-3 (PMC4236470; doi:10.1186/s13071-014-0489-3)
Supplement: Additional file 1: Table S1. — Akaike Information Criterion (AIC) values for the exponential, gamma, Gompertz, and Weibull fits to larval survival data (* indicates the best fit). [file 13071_2014_489_MOESM1_ESM.docx]

**Table S1**. **Median survival times of *An. gambiae* s.s. larvae at different environmental temperatures.**

| **Temperature (°C)** | **Total number of larvae exposed** | **Median survival (days) (95% C.I.)** |
| --- | --- | --- |
| 23±1 | 639 | ND* |
| 27±1 | 589 | ND* |
| 31±1 | 638 | ND* (9, ND) |
| 35±1 | 636 | 8 (8, 8) |

*ND: Not determined. Median survival defines the time point at which the survivorship curve crosses 0.5, or at which 50% of the sample is expected to survive. In this case, the survival function did not cross 0.5, and the median survival cannot be calculated.
