# Supplementary material for: Temperature during larval development and adult maintenance influences the survival of Anopheles gambiae s.s
Source: Parasit Vectors. 2014 Nov 5;7:489. doi: 10.1186/s13071-014-0489-3 (PMC4236470; doi:10.1186/s13071-014-0489-3)
Supplement: Additional file 2: Table S2. — Akaike Information Criterion (AIC) values for the exponential, gamma, Gompertz, and Weibull fits to adult survival data (* indicates the best fit). [file 13071_2014_489_MOESM2_ESM.docx]

**Table S2. Two-group comparisons and overall trend of the effect of larval environmental temperature on *An. gambiae* s.s. larval survival.**

| **Test statistic** | **27±1°C (with respect to 23°C)** | **31±1°C (with respect to 27°C)** | **35±1°C (with respect to 23°C)** | **35±1°C (with respect to 27°C)** | **35±1°C (with respect to 31°C)** | **Overall effect of temperature on larval survival** | |
| --- | --- | --- | --- | --- | --- | --- | --- |
| Mantel-Cox test | 28.97 | 29.63 | 642.10 | 932.70 | 798.60 | Log-rank test | 1510.00 |
| p-value | <0.001 | <0.001 | <0.001 | <0.001 | <0.001 | p-value | <0.001 |

* The comparison between 31°C and 23°C generated partly indistinguishable data, which did not allow us to perform a meaningful statistical test.
