# Supplementary material for: Temperature during larval development and adult maintenance influences the survival of Anopheles gambiae s.s
Source: Parasit Vectors. 2014 Nov 5;7:489. doi: 10.1186/s13071-014-0489-3 (PMC4236470; doi:10.1186/s13071-014-0489-3)
Supplement: Additional file 3: Table S3. — AICc values for the exponential, gamma, Gompertz, and Weibull fits to adult survival data, subdivided by larval temperature (* indicates the best fit, ‡ indicates where the Gompertz fit is not significantly worse than the best fit). [file 13071_2014_489_MOESM3_ESM.docx]

**Table S3. Median survival times of *An. gambiae* s.s. adults at different environmental temperatures.**

| **Temperature (°C)** | **Total number of larvae surviving to imagoes** | **Median survival (days) (95% C.I.)** |
| --- | --- | --- |
| 23±1 | 103 | ND* |
| 27±1 | 120 | 31 (30, 33) |
| 31±1 | 89 | 25 (24, 25) |

*ND: Not determined. Median survival defines the time point at which the survivorship curve crosses 0.5, or at which 50% of the sample is expected to survive. In this case, the survival function did not cross 0.5, and the median survival cannot be calculated.
