# Supplementary figures and images for: Temperature during larval development and adult maintenance influences the survival of Anopheles gambiae s.s
Source: Parasit Vectors. 2014 Nov 5;7:489. doi: 10.1186/s13071-014-0489-3 (PMC4236470; doi:10.1186/s13071-014-0489-3)

Supplementary figure s1.

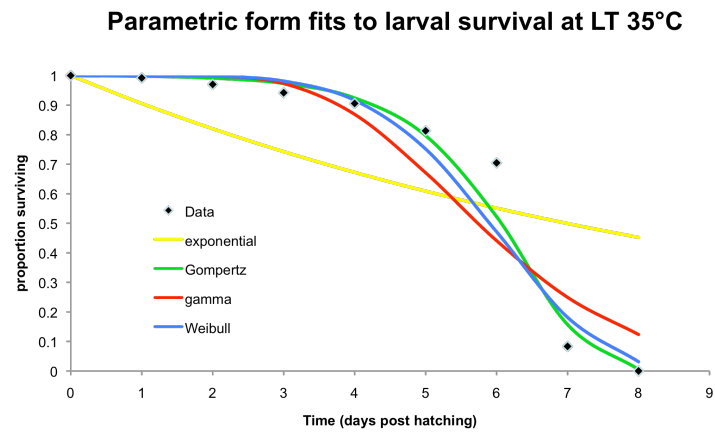

Supplement: Additional file 4: Figure S1. — Parametric fitting. An example of the fitting of four parametric survival functions (exponential (yellow), Gompertz (green), gamma (red), and Weibull (blue)) to larval survival data at environmental temperature 35°C. [file 13071_2014_489_MOESM4_ESM.pdf]

Supplementary figure s2.

Gompertz fit to larval survival curves

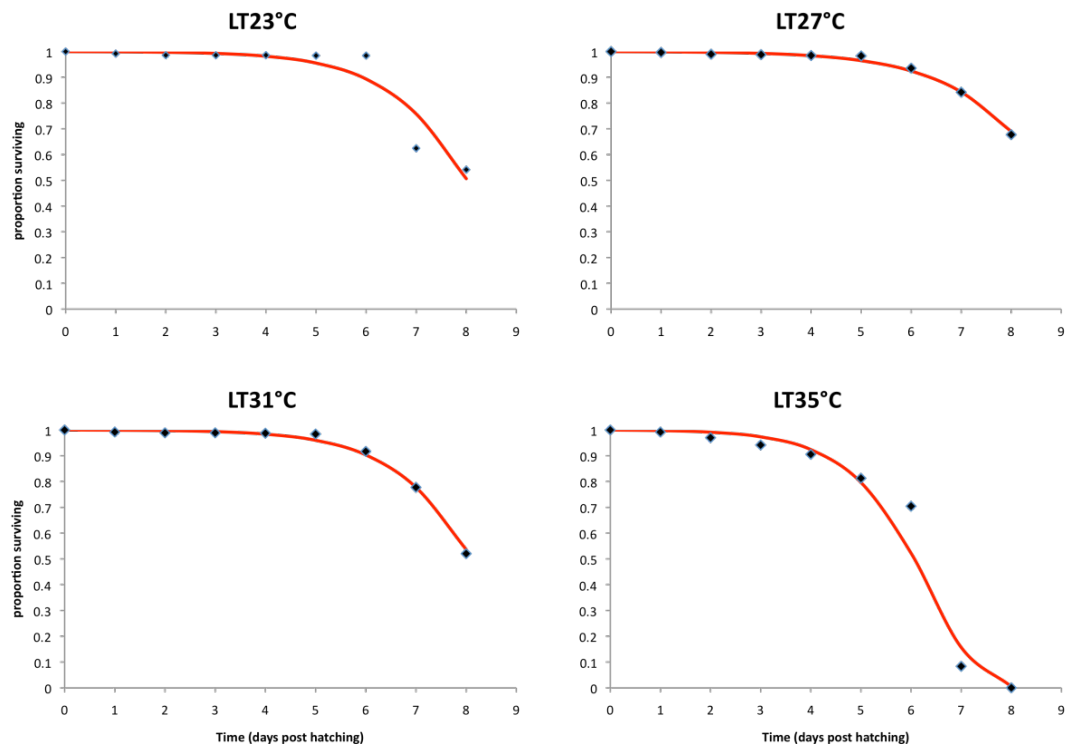

Supplement: Additional file 8: Figure S2. — Gompertz fits to larval survival data. The Gompertz survival functions (red) are shown alongside the larval survival data at all environmental temperatures (23°C, 27°C, 31°C, and 35°C) to which they were fitted. [file 13071_2014_489_MOESM8_ESM.pdf]

Supplementary figure s3. Gompertz fit to adult survival curves

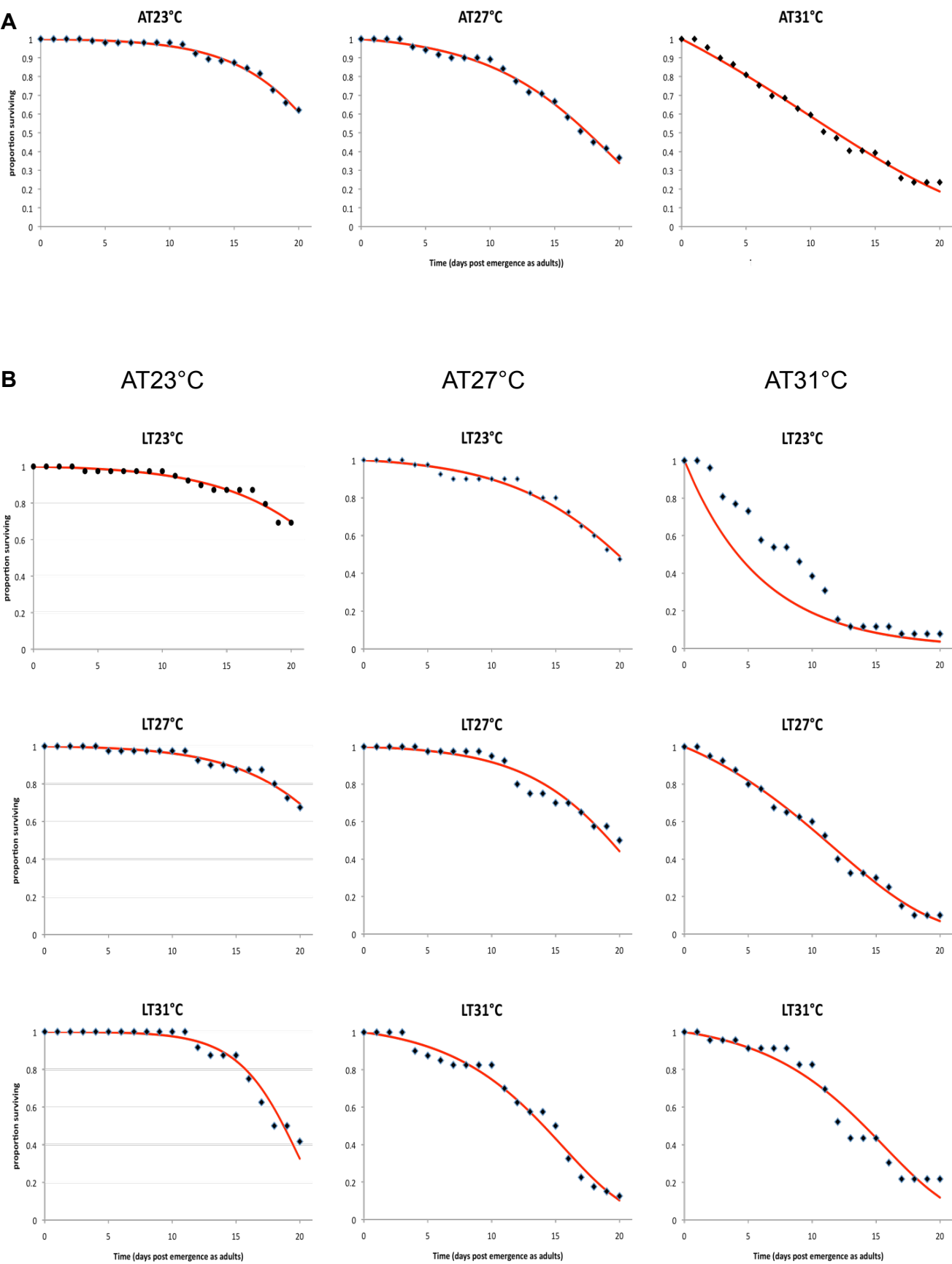

Supplement: Additional file 10: Figure S3. — Gompertz fits to adult survival data. (A). The Gompertz survival functions (red) are shown alongside the adult survival data at all adult temperatures (23°C, 27°C, 31°C) to which they were fitted. (B). The Gompertz survival functions (red) are shown alongside the adult survival data at all combinations of larval and adult temperatures to which they were fitted. [file 13071_2014_489_MOESM10_ESM.pdf]

Supplementary Figure s5.

Gompertz hazard function fit to larval mortality data

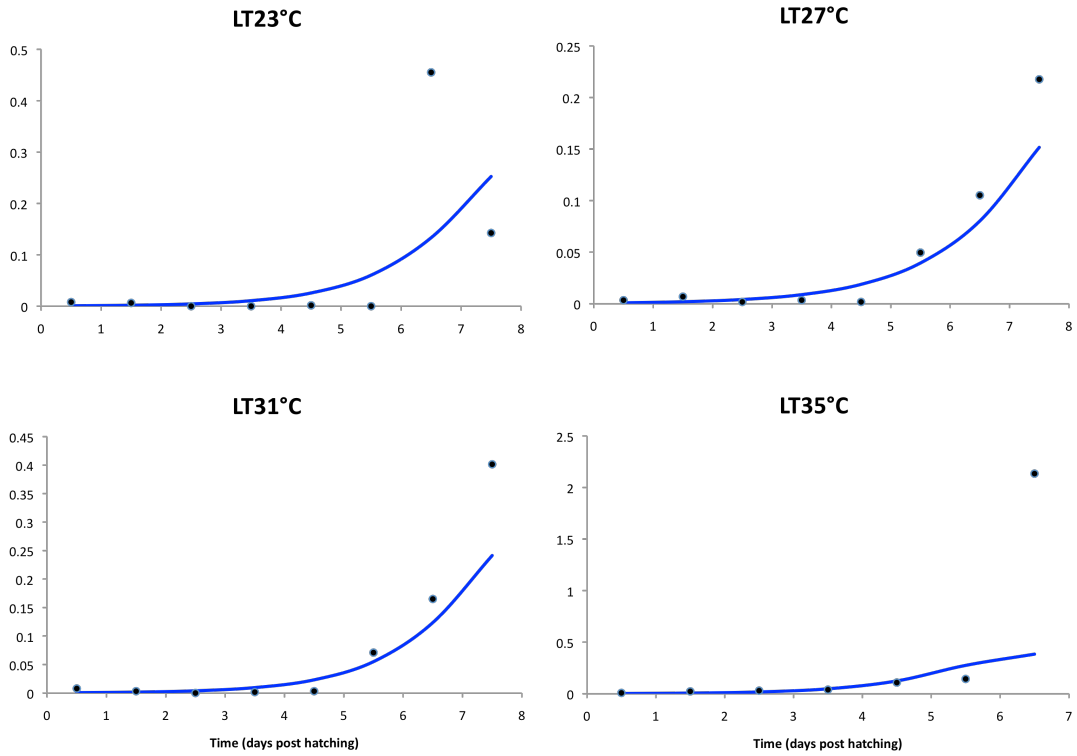

Supplement: Additional file 12: Figure S5. — Best-fit Gompertz survival function plotted against larval survival data. The Gompertz functions (blue) are shown alongside the larval survival data at all environmental temperatures (23°C, 27°C, 31°C, and 35°C). [file 13071_2014_489_MOESM12_ESM.pdf]

Supplementary Figure s6.

Gompertz hazard function fit to larval mortality data

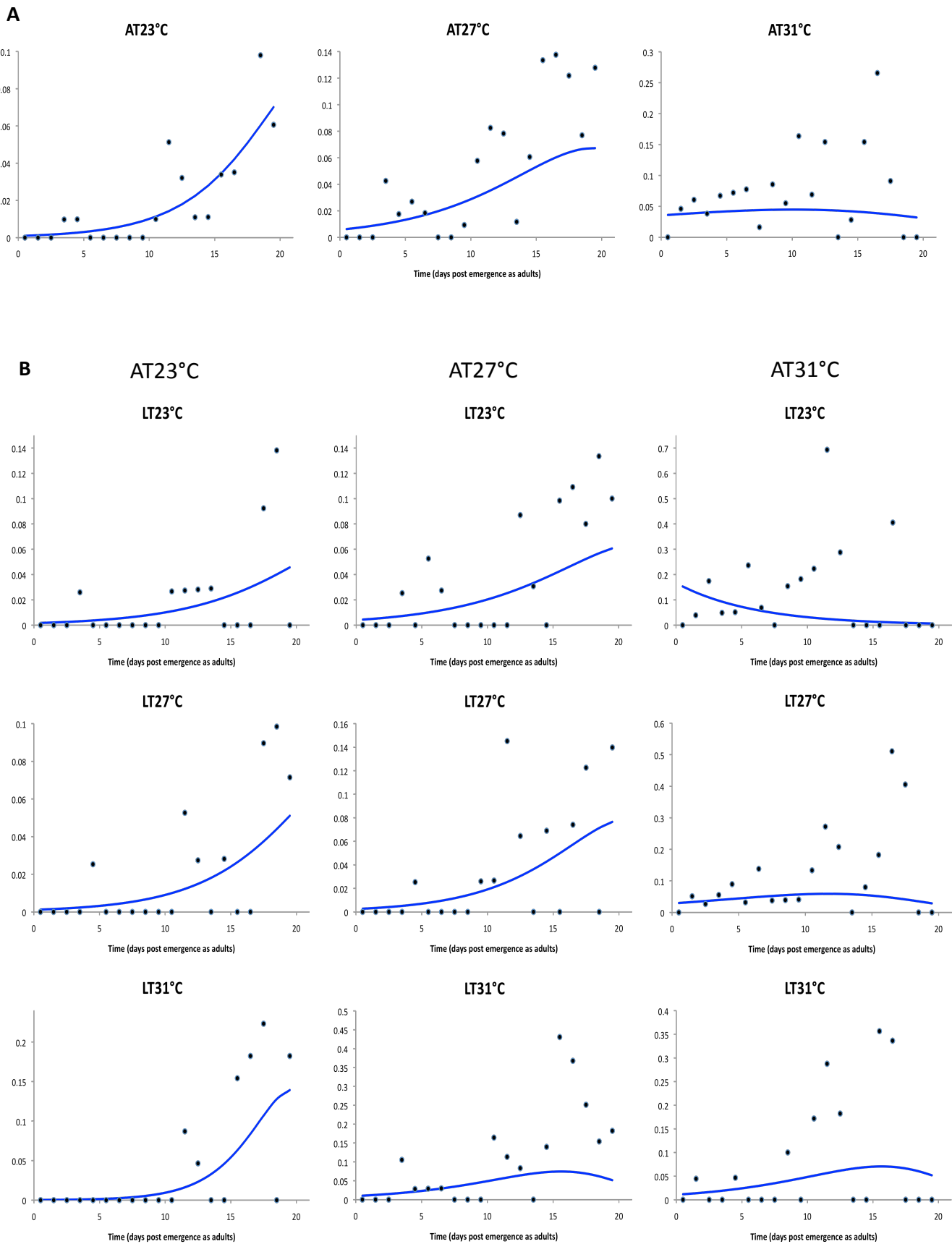

Supplement: Additional file 13: Figure S6. — Gompertz survival function plotted alongside adult mortality data. (A). The Gompertz functions (blue) are shown alongside the adult mortality data at all adult temperatures (23°C, 27°C, 31°C). (B). The Gompertz functions (blue) are shown alongside the adult mortality data at all combinations of larval and adult temperatures. [file 13071_2014_489_MOESM13_ESM.pdf]

Supplementary Figure s7.

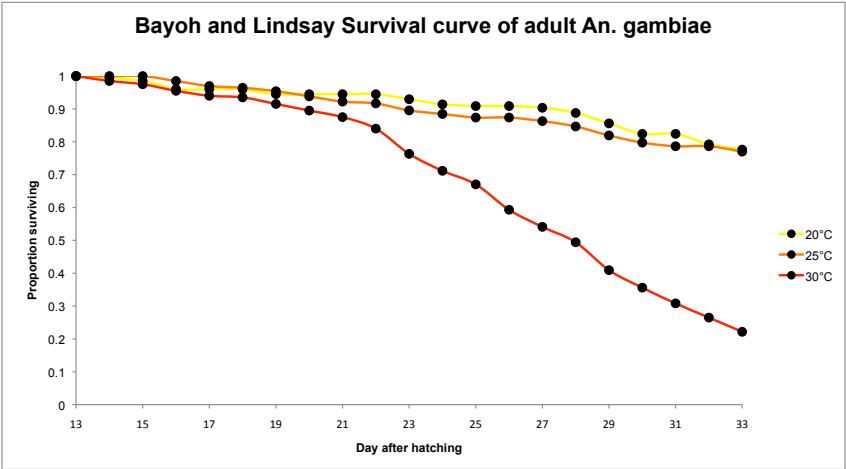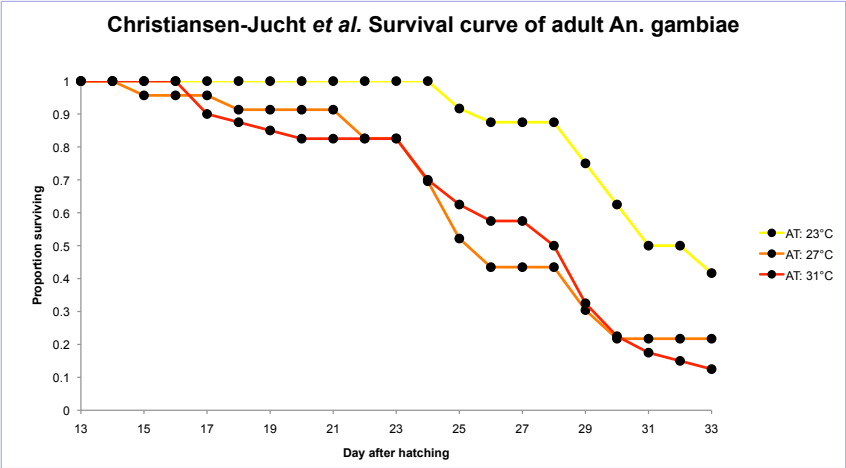

Supplement: Additional file 14: Figure S7. — Comparison of survival curves with those generated by Bayoh and Lindsay (30). (A). Survival curves by Bayoh and Lindsay, larval temperature 26°C, 80% RH. Adult survival at environmental temperature 20°C (yellow), 25°C (orange), and 30°C (red). (B). Survival curves with data from this study, larval temperature 27°C, 75% RH. Adult survival at environmental temperature 23°C (yellow), 27°C (orange), 31°C (red). [file 13071_2014_489_MOESM14_ESM.pdf]
