# Supplementary material for: Temperature during larval development and adult maintenance influences the survival of Anopheles gambiae s.s
Source: Parasit Vectors. 2014 Nov 5;7:489. doi: 10.1186/s13071-014-0489-3 (PMC4236470; doi:10.1186/s13071-014-0489-3)
Supplement: Additional file 5: Table S4. — Median survival times of An. gambiae s.s. larvae at different environmental temperatures. *ND: Not determined. Median survival defines the time point at which the survivorship curve crosses 0.5, or at which 50% of the sample is expected to survive. In this case, the survival function did not cross 0.5, and the median survival cannot be calculated. [file 13071_2014_489_MOESM5_ESM.docx]

**Table S4. Two-group comparisons and overall trend of the effect of adult environmental temperature on *An. gambiae* s.s. adult survival.**

| **Test statistic** | **27°C±1 (with respect to 23°C)** | **31°C±1 (with respect to 23°C)** | **31°C±1 (with respect to 27°C)** | **Overall effect of temperature on adult survival** | |
| --- | --- | --- | --- | --- | --- |
| Mantel-Cox test | 17.95 | 92.40 | 37.23 | Log-rank test | 102.30 |
| p-value | <0.001 | <0.001 | <0.001 | p-value | <0.001 |
