# Supplementary material for: Temperature during larval development and adult maintenance influences the survival of Anopheles gambiae s.s
Source: Parasit Vectors. 2014 Nov 5;7:489. doi: 10.1186/s13071-014-0489-3 (PMC4236470; doi:10.1186/s13071-014-0489-3)
Supplement: Additional file 6: Table S5. — Two-group comparisons and overall trend of the effect of larval environmental temperature on An. gambiae s.s. larval survival. *The comparison between 31°C and 23°C generated partly indistinguishable data, which did not allow us to perform a meaningful statistical test. [file 13071_2014_489_MOESM6_ESM.docx]

**Table S5.** **Akaike Information Criterion (AIC) values for the exponential, gamma, Gompertz, and Weibull fits to larval survival data (* indicates the best fit).**

| **Larval temperature** | **Parametric curve** | **AIC value** |
| --- | --- | --- |
| **23°C** | exponential | 3075.58 |
|  | gamma | 2640.55 |
|  | Gompertz | 2473.3 * |
|  | Weibull | 2560.13 |
| **27°C** | exponential | 2202.65 |
|  | gamma | 1990.12 |
|  | Gompertz | 1923.87 * |
|  | Weibull | 1964.27 |
| **31°C** | exponential | 2926.08 |
|  | gamma | 2531.45 |
|  | Gompertz | 2374.34 * |
|  | Weibull | 2459.54 |
| **35°C** | exponential | 4725.81 |
|  | gamma | 3360.68 |
|  | Gompertz | 2835.85 * |
|  | Weibull | 3047.92 |
