# Supplementary material for: Temperature during larval development and adult maintenance influences the survival of Anopheles gambiae s.s
Source: Parasit Vectors. 2014 Nov 5;7:489. doi: 10.1186/s13071-014-0489-3 (PMC4236470; doi:10.1186/s13071-014-0489-3)
Supplement: Additional file 7: Table S6. — Median survival times of An. gambiae s.s. adults at different environmental temperatures. *ND: Not determined. Median survival defines the time point at which the survivorship curve crosses 0.5, or at which 50% of the sample is expected to survive. In this case, the survival function did not cross 0.5, and the median survival cannot be calculated. [file 13071_2014_489_MOESM7_ESM.docx]

**Table S6. Akaike Information Criterion (AIC) values for the exponential, gamma, Gompertz, and Weibull fits to adult survival data (* indicates the best fit).**

| **Adult temperature** | **Parametric curve** | **AIC value** |
| --- | --- | --- |
| **23°C** | exponential | 1123.14 |
|  | gamma | 1034.29 |
|  | Gompertz | 1023.79 * |
|  | Weibull | 1029.24 |
| **27°C** | exponential | 2134.52 |
|  | gamma | 1998.65 |
|  | Gompertz | 1990.06 * |
|  | Weibull | 1992.33 |
| **31°C** | exponential | 1966.31 |
|  | gamma | 1928.30 * |
|  | Gompertz | 1934.92 |
|  | Weibull | 1928.94 |
