# Supplementary material for: Temperature during larval development and adult maintenance influences the survival of Anopheles gambiae s.s
Source: Parasit Vectors. 2014 Nov 5;7:489. doi: 10.1186/s13071-014-0489-3 (PMC4236470; doi:10.1186/s13071-014-0489-3)
Supplement: Additional file 9: Table S7. — Two-group comparisons and overall trend of the effect of adult environmental temperature on An. gambiae s.s. adult survival. [file 13071_2014_489_MOESM9_ESM.docx]

**Table S7. AICc values for the exponential, gamma, Gompertz, and Weibull fits to adult survival data, subdivided by larval temperature (* indicates the best fit, ^‡^** **indicates where the Gompertz fit is not significantly worse than the best fit).**

| **Adult temperature** | **Larval temperature** | **Parametric curve** | **AICc value** |
| --- | --- | --- | --- |
| **23°C** | **23°C** | exponential | 410.32 |
|  |  | gamma | 395.2 |
|  |  | Gompertz | 392.28 * |
|  |  | Weibull | 394.24 |
|  | **27°C** | exponential | 398.36 |
|  |  | gamma | 378.09 |
|  |  | Gompertz | 375.73 * |
|  |  | Weibull | 377.12 |
|  | **31°C** | exponential | 308.01 |
|  |  | gamma | 234.92 * |
|  |  | Gompertz | 239.62 |
|  |  | Weibull | 236.42 |
| **27°C** | **23°C** | exponential | 619.1 |
|  |  | gamma | 592.19 |
|  |  | Gompertz | 588.35 * |
|  |  | Weibull | 590.66 |
|  | **27°C** | exponential | 594.63 |
|  |  | gamma | 539.22 * |
|  |  | Gompertz | 544.83 |
|  |  | Weibull | 539.76 |
|  | **31°C** | exponential | 802.8 |
|  |  | gamma | 730.66 |
|  |  | Gompertz | 717.53 * |
|  |  | Weibull | 723.72 |
| **31°C** | **23°C** | exponential | 756.57 |
|  |  | gamma | 752.92 * |
|  |  | Gompertz | 758.79 |
|  |  | Weibull | 754.35 |
|  | **27°C** | exponential | 831.16 |
|  |  | gamma | 787.32 |
|  |  | Gompertz | 785.38 **^‡^** |
|  |  | Weibull | 784.87 * |
|  | **31°C** | exponential | 460.54 |
|  |  | gamma | 418.8 |
|  |  | Gompertz | 416.28 **^‡^** |
|  |  | Weibull | 415.49 * |
