# Supplementary material for: Temperature during larval development and adult maintenance influences the survival of Anopheles gambiae s.s
Source: Parasit Vectors. 2014 Nov 5;7:489. doi: 10.1186/s13071-014-0489-3 (PMC4236470; doi:10.1186/s13071-014-0489-3)
Supplement: Additional file 11: Figure S4. — Values of the Gompertz survival function parameters, λ and θ. (A). Parameters for the Gompertz survival function fitted to the larval survival data at each larval temperature are shown with their 95% confidence intervals (CI). (B). Parameters for the Gompertz survival function fitted to the adult survival data at each adult temperature are shown with their 95% CI. (C). Parameters for the Gompertz survival function fitted to the adult survival data at each combination of larval and adult temperatures are shown with their 95% CI. [file 13071_2014_489_MOESM11_ESM.pdf]

Supplementary figure s4

Gompertz parameters' values with respect to larval and adult environmental temperatures

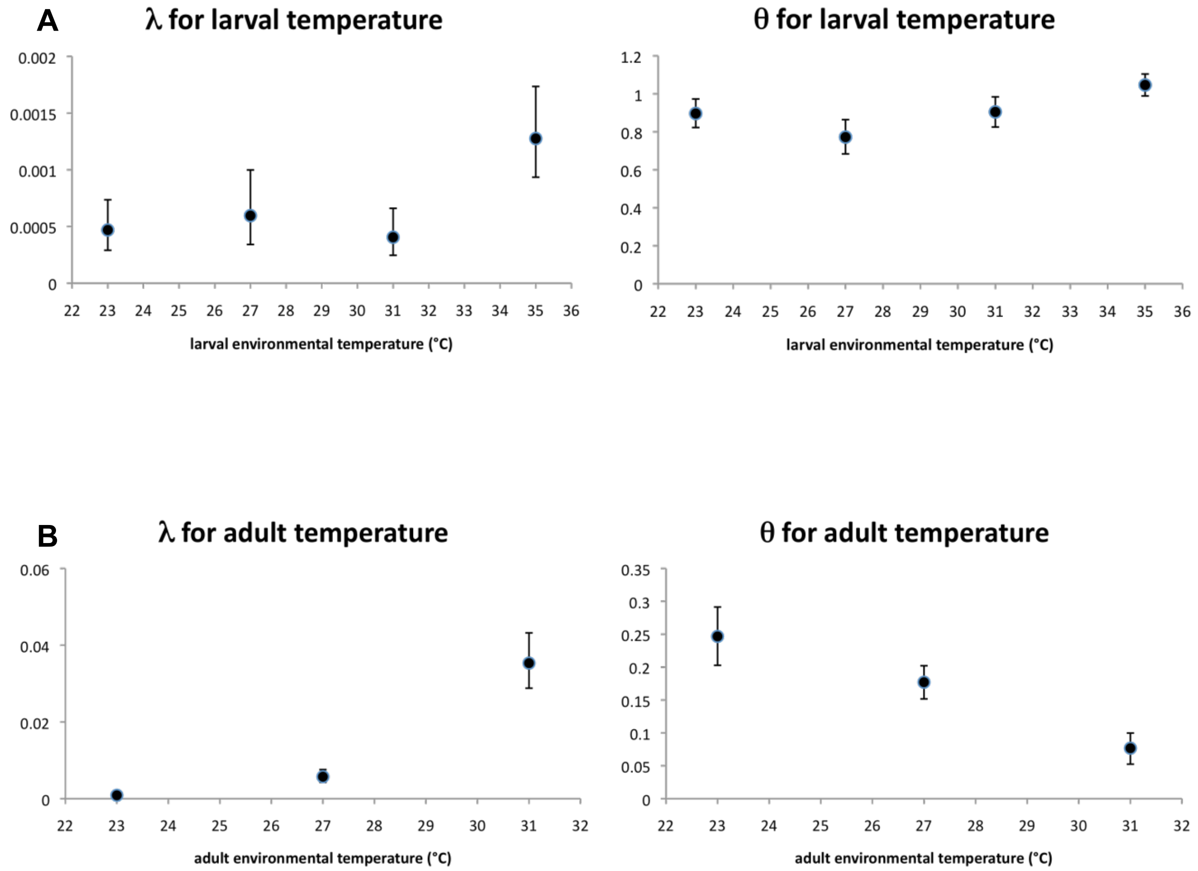

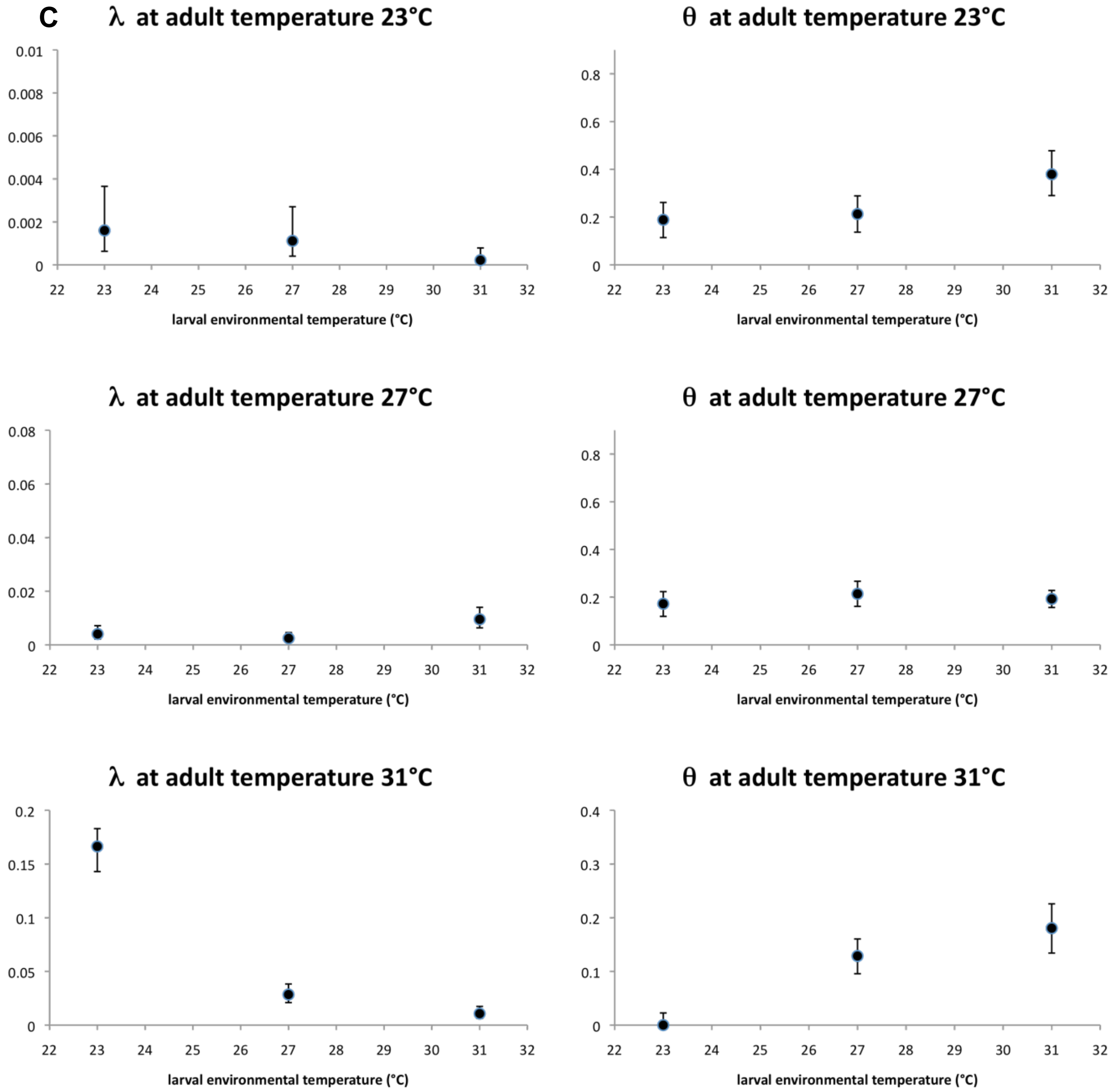
C
λ at adult temperature 23°C
θ at adult temperature 23°C
λ at adult temperature 27°C
θ at adult temperature 27°C
λ at adult temperature 31°C
θ at adult temperature 31°C
larval environmental temperature (°C)
